# Preparation data of the bromodomains BRD3(1), BRD3(2), BRD4(1), and BRPF1B and crystallization of BRD4(1)-inhibitor complexes

**DOI:** 10.1016/j.dib.2016.04.009

**Published:** 2016-04-11

**Authors:** Martin Hügle, Xavier Lucas, Gerhard Weitzel, Dmytro Ostrovskyi, Bernhard Breit, Stefan Gerhardt, Karin Schmidtkunz, Manfred Jung, Roland Schüle, Oliver Einsle, Stefan Günther, Daniel Wohlwend

**Affiliations:** a Albert-Ludwigs-Universität Freiburg, Institut für Biochemie, Albertstr. 21, D-79104 Freiburg, Germany; b College of Life Sciences, Division of Biological Chemistry and Drug Discovery, University of Dundee, James Black Centre, Dow Street, Dundee DD1 5EH, United Kingdom; c Albert-Ludwigs-Universität Freiburg, Institut für Organische Chemie, Albertstr. 21, D-79104 Freiburg, Germany; d Albert-Ludwigs-Universität Freiburg, Institut für Pharmazeutische Wissenschaften, Albertstr. 25, D-79104 Freiburg, Germany; e Urologische Klinik/Frauenklinik, Klinikum der Universität Freiburg and BIOSS Centre for Biological Signalling Studies, Freiburg, Germany; f Albert-Ludwigs-Universität Freiburg, Institut für Pharmazeutische Wissenschaften, Hermann-Herder-Str. 8, D-79104 Freiburg, Germany

**Keywords:** Epigenetics, Bromodomains, Drug discovery, X-ray crystallography

## Abstract

This article presents detailed purification procedures for the bromodomains BRD3(1), BRD3(2), BRD4(1), and BRPF1B. In addition we provide crystallization protocols for apo BRD4(1) and BRD4(1) in complex with numerous inhibitors. The protocols described here were successfully applied to obtain affinity data by isothermal titration calorimetry (ITC) and by differential scanning fluorimetry (DSF) as well as structural characterizations of BRD4(1) inhibitor complexes (PDB codes: PDB: 4LYI, PDB: 4LZS, PDB: 4LYW, PDB: 4LZR, PDB: 4LYS, PDB: 5D24, PDB: 5D25, PDB: 5D26, PDB: 5D3H, PDB: 5D3J, PDB: 5D3L, PDB: 5D3N, PDB: 5D3P, PDB: 5D3R, PDB: 5D3S, PDB: 5D3T). These data have been reported previously and are discussed in more detail elsewhere [1], [2].

**Table:** 

Subject area	Chemistry, Biology
More specific subject area	Structural biology, drug discovery
Type of data	Table, chromatograms, crystal images
How data was acquired	Chromatograms were acquired on Äkta Prime Plus systems, crystal images were acquired with a Zeiss SteREO Discovery 2.0 microscope equipped with a Canon EOS 600D CCD camera
Data format	Processed
Experimental factors	All proteins were produced heterologously in *E. coli*.
Experimental features	Proteins were isolated by IMAC via His_6_-tag followed by tag cleavage with TEV and polishing via size exclusion chromatography. Proteins were concentrated for crystallization to 10 mg/ml and supplied with ligands at a concentration of 2 mm.
Data source location	Institut für Biochemie, Albert-Ludwigs-Universität Freiburg, Albertstraße 21, D-79104 Freiburg, Germany
Data accessibility	Data is within this article. Structures were deposited in the PDB with the following PDB codes: PDB: 4LYI, PDB: 4LZS, PDB: 4LYW, PDB: 4LZR, PDB: 4LYS, PDB: 5D24, PDB: 5D25, PDB: 5D26, PDB: 5D3H, PDB: 5D3J, PDB: 5D3L, PDB: 5D3N, PDB: 5D3P, PDB: 5D3R, PDB: 5D3S, PDB: 5D3T

**Value of the data**

• Provides details on purification and crystallization of BRD4(1).
• Provides details on co-crystallization strategies of BRD4(1) with inhibitors.
• Provides details on purification of BRD3(1), BRD3(2), and BRPF1B.
• Data shown here may serve as benchmarks for other groups working with bromodomains.

## 1 Data

**Fig. 1:**
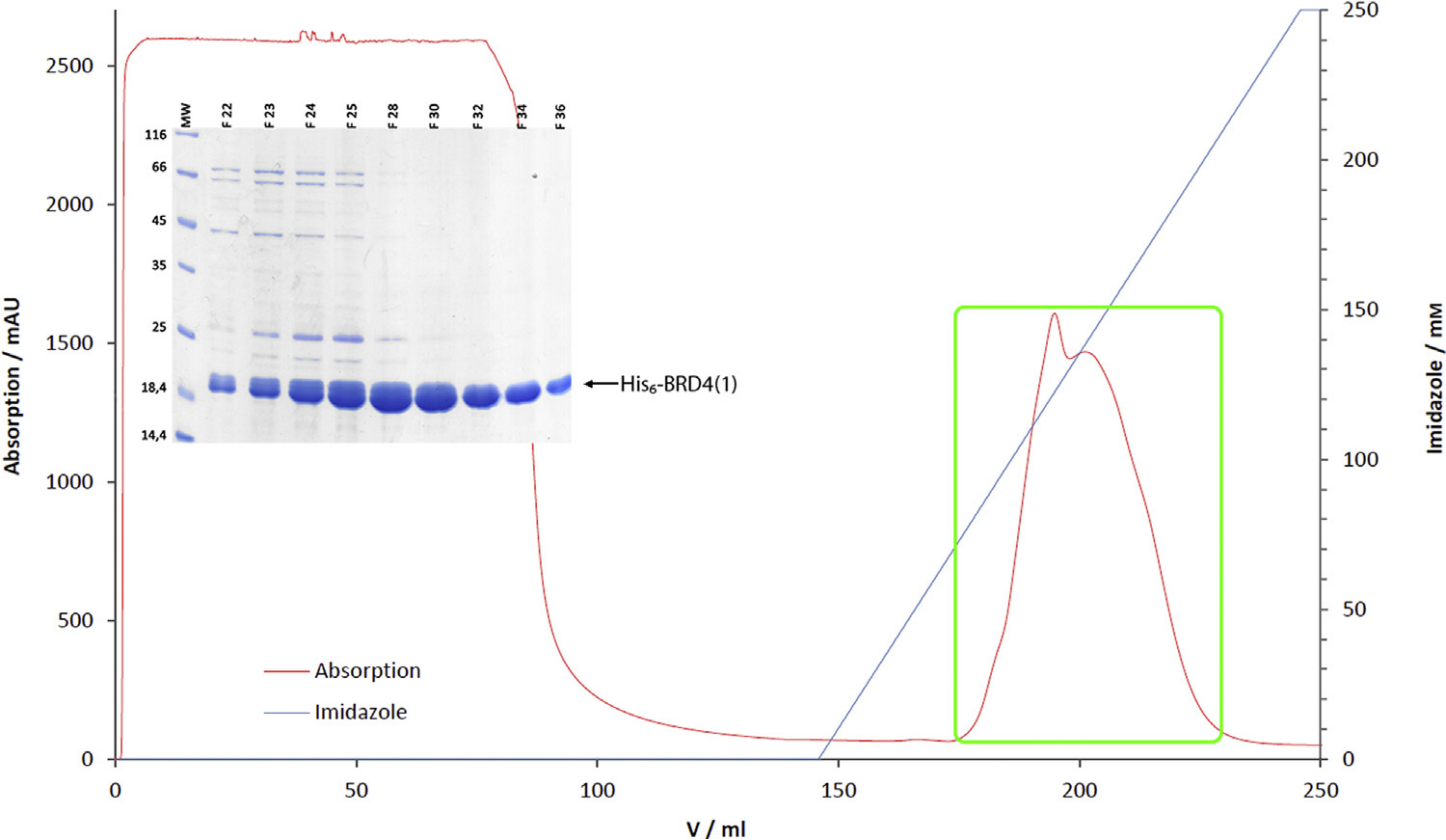
Purification chromatogram of His_6_-BRD4(1) via Ni-IMAC. The red curve shows the absorption at 280 nm in mAU, the blue curve represents the concentration of imidazole. (SDS-Gel) SDS-Gel of the framed peak. Bands of His_6_-BRD4(1) are marked with a black arrow, molecular weights of the marker are as indicated and the F-numbers above the gel refer to the fractions of the framed peak.

**Fig. 2:**
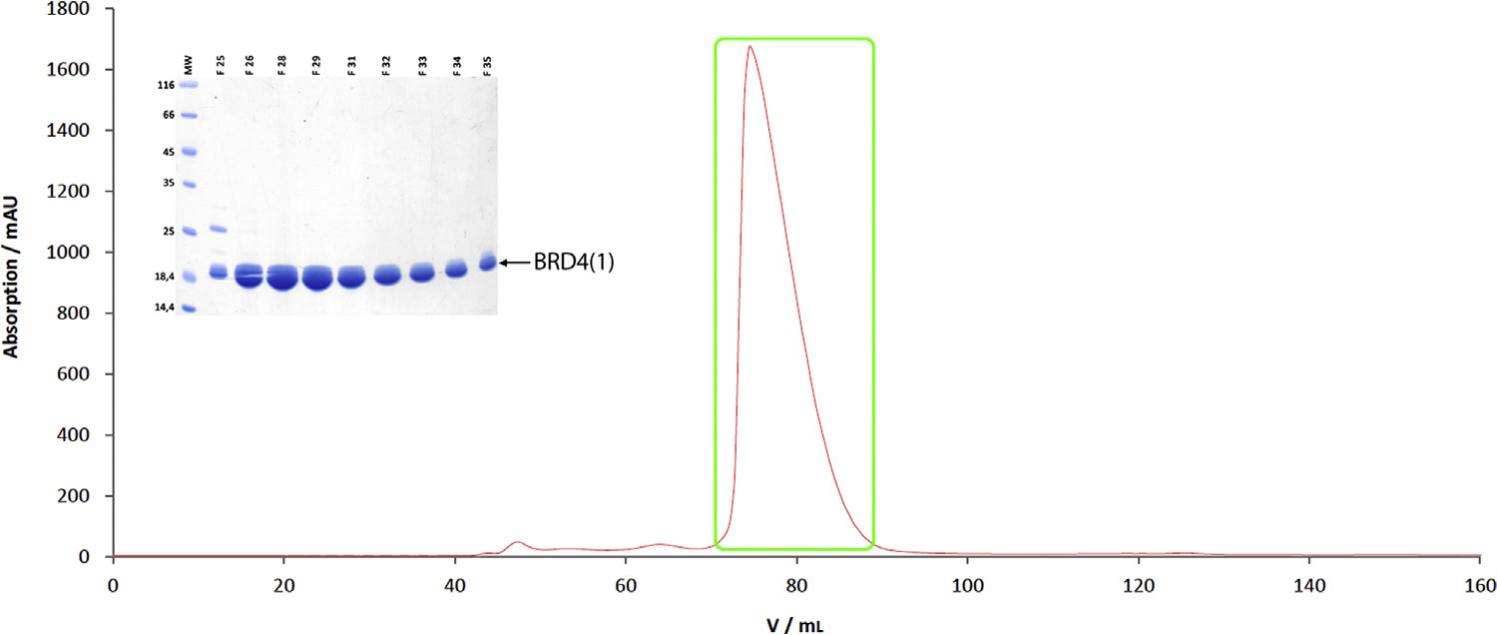
Purification chromatogram of BRD4(1) via size exclusion column. The red curve shows the absorption at 280 nm in mAU. (SDS-Gel) SDS-Gel of the framed peak. Bands of BRD4(1) are marked with a black arrow, molecular weights of the marker are as indicated and the F-numbers above the gel correspond to the fractions of the framed peak.

**Fig. 3:**
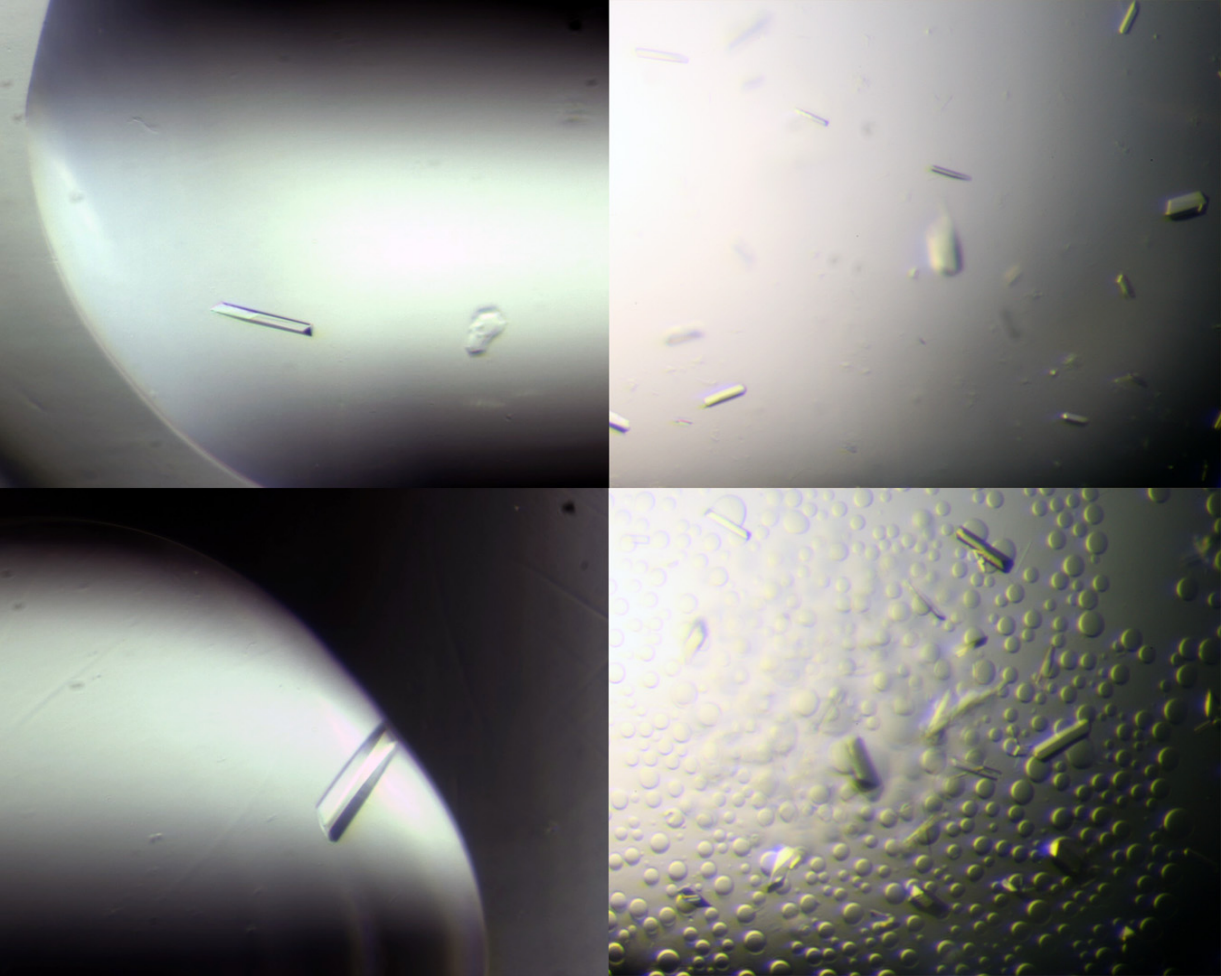
BRD4(1) crystals with various ligands. Initial drop size was always 400 nl. Crystal shapes differed only slightly even with different ligands and crystallization conditions. All crystals shared the space group P2_1_ 2_1_ 2_1_ yet with differing cell axes.

**Fig. 4:**
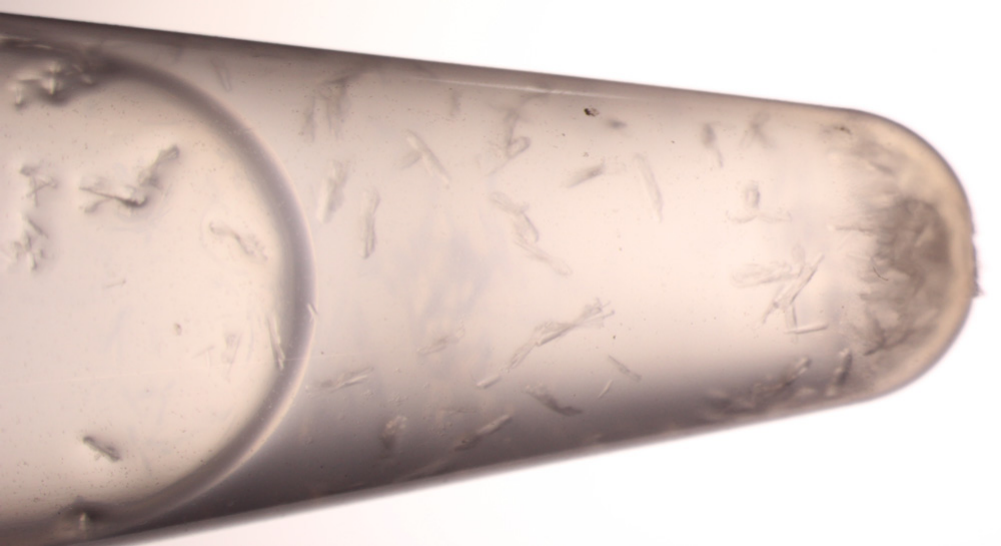
Spontaneous BRD4(1) crystal formation in reaction tube. BRD4(1) (20 mg/ml in crystallization buffer) crystals grown in a 1.5 ml reaction tube at 12 °C after two weeks.

We present a detailed strategy for the heterologous overproduction and purification of BRD4(1) including chromatograms and SDS-gels, shown in Fig. 1, Fig. 2. The data allowed for the characterization of 25 inhibitors for bromodomains with a special focus on BRD4(1), published in [1] and [2]. The protocol shown here was also used for the preparation of BRPF1B, BRD3(1) and BRD3(2) to determine their affinities to various novel inhibitors (see [1] for original affinity data). In addition, we provide photographs of crystals of apo BRD4(1) (Fig. 4) and BRD4(1) in complex with several inhibitors (Fig. 3).

For 14 inhibitors we obtained high resolution X-ray structures in complex with BRD4(1) [1], [2]. Structure formulas, SMILES strings and pdb ID codes are summarized in Supplementary Table 1.

## 2 Experimental design, materials and methods

### 2.1 Protein preparation

The plasmids for protein production are gifts from Nicola Burgess-Brown, purchasable via Addgene (Addgene plasmids # 38941, # 53620, # 38940, # 38943). All constructs share the vector backbone pNIC28-Bsa4 with a Kanamycin resistance gene. They encode for an N-terminal His_6_-tag followed by a TEV-cleavage site N-terminal of the target protein.

Chemically competent BL21 (DE3) cells were transformed with the constructs and plated on LB-Agar plates with Kanamycin (50 µg/ml). Single colonies were picked for preparatory cultures (LB media, 50 µg/ml Kanamycin, 37 °C, overnight). The preparatory cultures (10 ml) were then used to inoculate TB media (1 L, 50 µg/ml Kanamycin, 37 °C). At an OD_600_=2.5 the cells were cooled down to 20 °C and at an OD_600_=3 induced with IPTG (0.1 mM). Cells were harvested by centrifugation (5000x*g*, 15 min, 4 °C, JLA8.1000 rotor, Beckman Coulter Avanti J-26 XP) after 18 h. The cell pellets were re-suspended in lysis buffer (3 ml per g cells, 50 mM HEPES/NaOH, pH 7.5 at 20 °C, 500 mM NaCl, 30 mM Imidazole) and lysed using a M-110P fluidizer (Microfluidics, UK). Each lysate was cleared by centrifugation (100,000x*g*, 45 min, 4 °C, JA-30.50Ti rotor, Beckman Coulter Avanti J-30I) followed by filtration (0.45 µm). The filtered supernatant was then loaded onto a Nickel affinity column (5 ml HisTrap, GE Healthcare, Germany, equilibrated with 20 ml lysis buffer) connected to an ÄKTAPrime plus system (GE Healthcare, Germany). Immobilized protein was washed with lysis buffer to baseline and eluted in a linear gradient of imidazole in lysis buffer (0 to 250 mM imidazole over 100 ml, Fig. 1). The eluate was fractionated and analyzed via SDS-PAGE. The eluted target proteins were pooled and cleaved with TEV protease (1:200 M ratio, 24 h, 4 °C). Cleaved proteins were analyzed via SDS-PAGE, and further purified via size exclusion chromatography (HiLoad Superdex 75 XK 26/60, GE Healthcare, Germany, equilibrated with 400 ml crystallization buffer, 10 mM HEPES/NaOH, pH 7.5 at 20 °C, 150 mM NaCl) on an ÄktaPrime plus system (Fig. 2). Eluted proteins were analyzed via SDS-PAGE and concentrated to 10 mg/ml (VIVASPIN TURBO 15, 10,000 MWCO, Sartorius, Germany).

### 2.2 Protein crystallization

Prior to crystallization the protein solutions were supplemented with inhibitor from a 100 mM stock solution in DMSO to a final inhibitor concentration of 2 mM Subsequently, precipitated protein was removed by centrifugation (16,000x*g*, 4 °C, 5 min, Centrifuge 5415 R, Eppendorf, Germany). Co-crystals with the described inhibitors were obtained in the Index HT Screen (Hampton Research, Aliso Viejo, USA) using 38% protein (10 mg/ml) and 62% reservoir solution. The drops (400 nl) were pipetted into an Intelli-Plate 96 low profile (Hampton Research, USA) with an Oryx Nano crystallization robot (Douglas Instruments, UK). Single crystals formed after one to five days at 20 °C (Fig. 3). Note, that BRD4(1) crystallizes spontaneously after two weeks in the final crystallization buffer at 10–12 °C in a reaction tube, if the concentration exceeds 20 mg/ml (Fig. 4).
