# Supplementary material for: Preparation data of the bromodomains BRD3(1), BRD3(2), BRD4(1), and BRPF1B and crystallization of BRD4(1)-inhibitor complexes
Source: Data Brief. 2016 Apr 11;7:1370–4. doi: 10.1016/j.dib.2016.04.009 (PMC4845076; doi:10.1016/j.dib.2016.04.009)
Supplement: Supplementary file 1 — Supplementary material [file mmc1.docx]

Supplementary Table 1: Inhibitors of bromodomains BRD4(1), BRD3(1), BRD3(2), and BRPF1. Numbers in brackets indicate the numbering in the original publication [1]. PDB ID codes are provided where available. Affinity data were published in the original publications [1] and [2].

| Inhibitor | Structure and SMILES | Reference | PDB Code |
| --- | --- | --- | --- |
| apo |  | 2 | 4LYI |
| XD1 |   OC1=CC=C2C3=C(C=C(OC)C(OC)=C3OC)CC[C@H](NC(C)=O)C2=CC1=O | 2 | 4LYS |
| XD14 (1) |   CCc1c(C(Nc2c(O)ccc(S(=O)(N(CC)CC)=O)c2)=O)[nH]c(C)c1C(C)=O | 1,2 | 4LYW |
| XD15 |   CC1=NOC(CNC(NC2=CC(S(=O)(N(CC)CC)=O)=CC=C2OC)=O)=C1 | 2 | - |
| XD16 |   ClC1=CC(CC2=NOC(CNC3=CC4=C(C=C3)NCC4=O)=N2)=CC=C1 | 2 | - |
| XD19 |   N[C@H]1CCN(C2=NC(C3=CC=CC=C3)=CC(N2)=O)C1 | 2 | - |
| XD24 |   O=C1NC(CCC(NC2=NNC=C2)=O)=NC3=CC=CC=C31 | 2 | - |
| XD25 |   OC(C(O1)=CC=C1C2=CC(NC)=NC3=C2C=CN3)=O | 2 | 4LZR |
| XD26 (14) |   O=C1C(OC)=CC=C2C3=C(C=C(OC)C(OC)=C3OC)CC[C@H](NC(C)=O)C2=C1 | 1 | 5D24 |
| XD27 (2) |   O=C(Nc1cc(OCC(N)=O)ccc1)c2[nH]c(C)c(C(C)=O)c2CC | 1 | 5D25 |
| XD28 (3) |   Cc1c(C(C)=O)c(C)[nH]c1C(Nc2cc(S(=O)(N(CC)CC)=O)ccc2O)=O | 1 | 5D26 |
| XD29 (4) |   Oc1ccc(S(=O)(N(CC)CC)=O)cc1NC(c2[nH]c(C)c(C(N)=O)c2CC)=O | 1 | 5D3H |
| XD31 (9) |   Oc1ccc(S(=O)(N(CC)CC)=O)cc1NC(c2[nH]c(C)c(C(CO)=O)c2CC)=O | 1 | - |
| XD32 (5) |   Oc1ccc(S(=O)(N2CCCC2)=O)cc1NC(c3[nH]c(C)c(C(C)=O)c3CC)=O | 1 | - |
| XD33 (6) |   Oc1ccc(S(=O)(N(CC)CC)=O)cc1NC(c2[nH]c(C)c(C(C)=O)c2C(C)C)=O | 1 | 5D3J |
| XD34 (11) |   O=C(Nc1cc(S(=O)(N(CC)CC)=O)ccc1)c2[nH]c(C)c(C(C)=O)c2CC | 1 | - |
| XD35 (13) |   O=C(Nc1cc(S(=O)(N(CC)CC)=O)c(Cl)cc1)c2[nH]c(C)c(C(C)=O)c2CC | 1 | 5D3L |
| XD39 (15) |   O=C(Nc1cc(S(=O)(N(CC)CC)=O)c(C)cc1O)c2[nH]c(C)c(C(C)=O)c2CC | 1 | - |
| XD40 (7) |   O=C(Nc1cc(S(=O)(NC2=CC=CC=C2OC)=O)ccc1O)c3[nH]c(C)c(C(C)=O)c3CC | 1 | 5D3N |
| XD41 (10) |   O=C(Nc1cc(S(=O)(NC)=O)ccc1C)c2[nH]c(C)c(C(C)=O)c2CC | 1 | 5D3P |
| XD42 (17) |   O=C(N1c2cc(S(=O)(NC)=O)ccc2CC1)c3[nH]c(C)c(C(C)=O)c3CC | 1 | 5D3R |
| XD44 (12) |   O=C(Nc1cc(S(=O)(N2CCCCCC2)=O)ccc1)c3[nH]c(C)c(C(C)=O)c3CCC | 1 | 5D3S |
| XD45 (8) |   O=C(Nc1cc(S(=O)(N2CCOCC2)=O)c(F)cc1)c3[nH]c(C)c(C(C)=O)c3CC | 1 | - |
| XD46 (18) |   O=C(Nc1cc(C(N(CC)CC)=O)ccc1)c2[nH]c(C)c(C(C)=O)c2CC | 1,2 | 4LZS |
| XD47 (16) |   O=C(NC)c1[nH]c(C)c(C(C)=O)c1CC | 1 | 5D3T |
